# Can a quality improvement project impact maternal and child health outcomes at scale in northern Ghana?

**DOI:** 10.1186/s12961-016-0115-2

**Published:** 2016-06-16

**Authors:** Kavita Singh, Paul Brodish, Ilene Speizer, Pierre Barker, Issac Amenga-Etego, Ireneous Dasoberi, Ernest Kanyoke, Eric A. Boadu, Elma Yabang, Sodzi Sodzi-Tettey

**Affiliations:** Department of Maternal and Child Health, Gillings School of Global Public Health, University of North Carolina at Chapel Hill, Chapel Hill, NC 27516 United States of America; Carolina Population Center, University of North Carolina at Chapel Hill, Chapel Hill, NC United States of America; The Institute for Healthcare Improvement (IHI), Cambridge, MA United States of America; The National Catholic Health Service, Accra, Ghana; The Institute for Healthcare Improvement (IHI), Accra, Ghana

**Keywords:** Child Health, Ghana, Impact evaluation, Maternal health, Scale-up, Quality improvement

## Abstract

**Background:**

Quality improvement (QI) interventions are becoming more common in low- and middle-income countries, yet few studies have presented impact evaluations of these approaches. In this paper, we present an impact evaluation of a scale-up phase of ‘Project Fives Alive!’, a QI intervention in Ghana that aims to improve maternal and child health outcomes. ‘Project Fives Alive!’ employed a QI methodology to recognize barriers to care-seeking and care provision at the facility level and then to identify, test and implement simple and low-cost local solutions that address the barriers.

**Methods:**

A quasi-experimental design, multivariable interrupted time series analysis, with data coming from 744 health facilities and controlling for potential confounding factors, was used to study the effect of the project. The key independent variables were the change categories (interventions implemented) and implementation phase – Wave 2a (early phase) versus Wave 2b (later phase). The outcomes studied were early antenatal care (ANC), skilled delivery, facility-level under-five mortality and attendance of underweight infants at child welfare clinics. We stratified the analysis by facility type, namely health posts, health centres and hospitals.

**Results:**

Several of the specific change categories were significantly associated with improved outcomes. For example, three of five change categories (early ANC, four or more ANC visits and skilled delivery/immediate postnatal care (PNC)) for health posts and two of five change categories (health education and triage) for hospitals were associated with increased skilled delivery. These change categories were associated with increases in skilled delivery varying from 28% to 58%. PNC changes for health posts and health centres were associated with greater attendance of underweight infants at child welfare clinics. The triage change category was associated with increased early antenatal care in hospitals. Intensity, the number of change categories tested, was associated with increased skilled delivery in health centres and reduced under-five mortality in hospitals.

**Conclusions:**

Using an innovative evaluation technique we determined that ‘Project Fives Alive!’ demonstrated impact at scale for the outcomes studied. The QI approach used by this project should be considered by other low- and middle-income countries in their efforts to improve maternal and child health.

## Background

Quality improvement (QI) approaches are increasingly being used in low- and middle-income countries in efforts to improve service delivery and health outcomes. Much of the literature on QI approaches in these settings focuses on documentation of implementation and process evaluation [1,2]. A few studies have described scale-up processes for QI interventions in Ecuador [3,4], India [5] and South Africa [6,7]. All of these studies provide valuable information to guide countries and projects. However, documenting the impact of such approaches, both during pilot stages and at scale, is also important. This paper presents an impact evaluation of the scale-up phase of ‘Project Fives Alive!’, a national QI intervention in Ghana that aimed to improve maternal and child health outcomes. The project was implemented by the National Catholic Health Service and Institute for Healthcare Improvement in collaboration with the Ghana Health Service. The project design has been described previously in detail [8], and a prior evaluation study documented the impact of the pilot phase of the project [9].

The objective of ‘Project Fives Alive!’ was to assist and accelerate Ghana’s efforts to achieve Millennium Development Goals (MDGs) 4 (reducing under-five mortality) and 5 (reducing maternal mortality). Though Ghana did not meet the targets for MDG-4 and MDG-5, large improvements were made. In 2013, Ghana reported having a maternal mortality ratio of 380 maternal deaths per 100,000 live births, a large decline from the estimate of 760 maternal deaths per 100,000 live births in 1990 [10]. In 2015, the under-five mortality rate was estimated at 62 under-five deaths per 1000 live births compared to 122/1000 in 1990 [11].

‘Project Fives Alive!’ began in July 2008 with an innovation and testing phase, Wave 1, which included 27 health facilities in Northern Ghana. These facilities were purposively selected to reflect a mix of government facilities and faith-based facilities, which are affiliated with a religious institution. Wave 1 provided an opportunity for the implementation team to develop a package of locally identified and tested change ideas (interventions) focused on improving care seeking and care giving for mothers and children. Following Wave 1, the project rapidly introduced the locally developed interventions (changes) through a subsequent scale-up phase, Wave 2, to all government and faith-based facilities in Northern Ghana. Wave 2 included over 800 health facilities and covered the time period of September 2009 to March 2013.

‘Project Fives Alive!’ used the Model for Improvement and its underlying QI approach of identifying gaps in performance and the process failures that led to those gaps. The next step is identifying and testing simple low cost change ideas (or interventions) that can be employed to address those failures [12]. The process of generating, testing and sharing those ideas is accelerated through the Institute for Healthcare Improvement’s Collaborative Breakthrough Series Model that brought multiple sub-district or facility QI teams together repeatedly to share knowledge for improving performance. These teams formed Improvement Collaborative Networks at the district level [13] within specific geographic locations. During Wave 1, QI teams were formed at the level of the facility, while in Wave 2 teams were formed at the sub-district level such that all health centres and health posts within a sub-district contributed team members to form one team. Due to the higher volume and higher acuity of patients in hospitals settings, each hospital in Wave 2 formed its own team. Both Waves used the same basic Breakthrough Series approach; the QI teams attended four Learning Sessions (structured workshops led by project staff) where they learned QI methods and had a chance to share progress and ideas with other QI teams. During the 4–6 months between the Learning Sessions, both Waves 1 and 2 included Activity Periods when QI teams conducted Plan-Do-Study-Act cycles, the primary mechanism for testing and implementing changes. These cycles involved small tests of changes followed by rapid evaluations and adaptions.

Though the basic approach was the same, there were some key differences in implementation between Waves 1 and 2. Due to the small scale of Wave 1 (n = 27) and focus on innovation and development of change packages, the project team spent considerable time coaching each QI team during the Activity Periods. However, due to the large numbers of facilities in Wave 2 and only a small increase in project staff, it was not feasible to continue with this programmatically intense strategy. Thus, district health staff undertook intensive training and performed more of the coaching activities under the close supervision of project staff.

The main objective of this paper is to determine whether ‘Project Fives Alive!’ influenced maternal and child health outcomes at scale. A secondary objective is to present a methodology of using facility-based routine health data for a large-scale impact evaluation.

## Methods

We employed a quasi-experimental design with a multivariable interrupted time series analysis controlling for potential confounding factors, to understand the impact of the intervention. Outcome data for this analysis are derived from data measured and reported by the facilities, while independent variables come from facility and program records. A specific programmatic decision was made to use routinely reported data rather than institute a parallel project data collection system, since the intervention was designed to be sustainable and scalable. To support this decision, a major effort was undertaken to improve the timeliness, completeness and accuracy of the data being submitted to and reported by the Ghana District Health Information Management System (DHIMS), a system whereby facilities complete monthly reports of key indicators and these are compiled at the district and national levels.

A total of 744 facilities were included in our analysis. Some newer facilities could not be included because of a lack of pre-intervention data. Since the intervention has the potential to differentially impact health outcomes by facility type and also due to different degrees of missing data, the analyses are stratified by facility type: health posts (or first level facility), health centres, and hospitals. Ethics review approval was obtained by the Ghana Health Service and the University of North Carolina at Chapel Hill.

### Outcome Data

This evaluation used data from January 2009 to March 2013. In the initial phase of Wave 2, facilities used paper-based forms to report on key outcome indicators in a system called DHIMS 1. These forms were compiled and entered at the district level and then electronically sent onwards to the national level. In January 2012, Ghana shifted to a complete electronic system, DHIMS 2, whereby facilities entered the data and submitted the forms directly to the national level.

The four outcome variables in this assessment were chosen based on relevance to the project, and three were also included in the Wave 1 impact evaluation. Each outcome variable studied and the exact metric we used to define the variable are described in Table 1. The maternal health variables are early antenatal care (ANC) and skilled delivery coverage. We were able to study coverage for skilled delivery because health facilities record births that occur both at home and in facilities. The child health outcomes included the percent of child welfare clinic (CWC) attendees who are underweight and facility-level under-five mortality (for hospitals only). Underweight is defined as low weight for age in comparison to WHO reference standards. Our definition encompasses both moderate (less than two standard deviations below the median of the reference) and severe underweight (less than three standard deviations below the median of the reference standard) [14]. We could not study neonatal and infant mortality because of data quality concerns stemming from changes in reporting from DHIMS 1 to DHIMS 2. Fewer facilities reported these mortality outcomes in the DHIMS 2.

**Table 1:** Outcome variables and their definition/metric

Outcome	Definition/Metric
Early antenatal care (ANC)	% of ANC registrants in the first trimester at the time of registration
	Numerator: number of ANC registrants in first trimester at registration
	Denominator: number of ANC registrants
Skilled delivery coverage	% of total deliveries that are attended by skilled personnel
	Numerator: number of total deliveries that are attended by skilled health personnel
	Denominator: number of total deliveries (both skilled and unskilled) from facility and non-facility settings
Underweight infants at child welfare clinics (CWC)	% of 1- to 11-month-old CWC attendees < 60% weight for age
	Numerator: number of 1- to 11-month-old CWC attendees who are moderately or severely underweight
	Denominator: total number of 1- to 11-month-old CWC attendees with weight checked
Under-five mortality	Facility-level mortality among children less than 5 years of age
	Numerator: number of deaths among children aged 0–59 months in hospitals
	Denominator: total number of hospital admissions of children aged 0–59 months

### Key independent variables

One of the two key independent variables was the interventions or change ideas implemented at a facility. For health centres and health posts, the change ideas were grouped into five categories – early pregnancy identification, four or more ANC visits, skilled delivery/immediate postnatal care (PNC), PNC on day 1 or 2, and PNC on day 6 or 7. Examples of these change interventions included community stakeholder meetings and registration of pregnant women by community volunteers for the early ANC; ANC defaulter tracing and visit time reduction for the four or more ANC visits; use of partographs and immediate checks of mother and newborn for the skilled delivery/immediate PNC; and home visits for both PNC interventions.

Hospitals had a separate set of change categories more suited to their patient loads and the presence of higher level staff. Hospital change categories were health education, targeting/engaging primary providers, training, triage and task shifting/nurse empowerment. The hospital changes were more broadly targeted than the health centre and health post changes since hospitals cover all types of services. Hospital changes were expected to improve maternal and child health by shortening visit times, prioritizing sick mothers and children, and improving communication between providers and pregnant women and mothers.

The other key independent variable was the implementation phase in which these improvement activities occurred – Wave 2a or Wave 2b. The earlier phase, Wave 2a, included the majority of facilities, whereas Wave 2b included the later set of facilities to engage in implementation. We study this variable to understand whether all facilities benefit equally or whether facilities that initiate implementation earlier, benefit more.

### Control variables

The facility-level control variables included in this analysis were the type of health facility (hospital, health centre or health post) and affiliation of the health facility (government or faith based). A dummy variable was also included to represent the project officer assigned to work with a particular QI team. We also included as control variables profession of the QI team leader and number of QI team members. Since health insurance, particularly Ghana’s National Health Insurance Scheme, may be a potential confounding factor, a monthly time varying health insurance control variable (which was measured as the percent of outpatients who had insurance) was included.

### Analysis

#### Descriptive analysis

In our descriptive analysis, we present a comparison of the pre-intervention, transition phase and post-intervention means of the outcome variables. The pre-intervention phase was defined by the project implementation team as the period of time before Learning Session 1, when QI teams were still learning the methodology of testing changes. The transition phase was the time period from Learning Session 1 to the end of Learning Session 2, which was considered a time when teams had just completed the training needed to fully implement the QI approach. The post-intervention or full saturation phase began at the very end of Activity Period 2 and was considered the cut-off point when the QI teams were expected to have the skills and knowledge to fully implement change ideas.

#### Missing outcome data

**Fig. 1:**
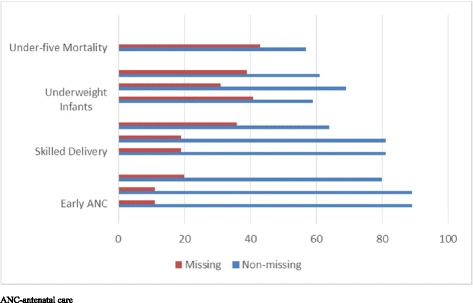
Percent of facilities with missing and non-missing data for health outcomes

The unit of observation for the outcome data was facility-months. Each facility had several months of data. The number of facility months varied by outcome because not all facilities reported on all outcomes, and some facilities were new and were not in existence during the early time points or did not report on a particular outcome at exactly time 1. In addition, missing data, which was defined as an outcome not reported in a particular month once a facility has initiated reporting, was also responsible for some of the differences. Furthermore, with Ghana’s change in facility-level reporting of outcomes in January 2012, some facilities no longer provided denominators needed for the skilled delivery outcome. For these facilities, we ended their observation interval in December 2011 for skilled delivery to avoid considering the data points from January 2012 onwards as missing. Figure 1 presents the amount of missing data both by facility and outcome. Hospitals and health centres had relatively low amounts of missing data for the maternal health outcomes, whereas health posts had slightly more missing for these outcomes. Missing data for attendance of underweight infants at CWCs varied from 31% for health centres to 41% for hospitals. Hospitals had 43% missing data for under-five mortality.

#### Multivariable time series analysis

To study the impact of ‘Project Fives Alive!’ on the outcomes, we employed a multivariable interrupted time series regression analysis. This type of analysis answers the question of whether an intervention is associated with a change in the underlying trend for the outcome of interest after controlling for key variables [15]. The methodology of using repeated or monthly observations from the same facilities both pre- and post-intervention offers a strong evaluation design [16]. Data came from the period of January 2009 to March 2013, and the first set of facilities did not reach full implementation until July 2010. It was thus possible to establish underlying trends using the pre-intervention and transition phase data. In this analysis each facility served as its own control with the pre-intervention trend compared to the post-intervention trend.

In our model, there are two key parameters that were of interest – the immediate impact of the change category and the longer term impact or change category trend. Adding the coefficients from these two parameters yields the overall effect of the change category. Our model also included a quadratic term to account for a potential non-linear trend. A detailed description of the regression model and equation are presented by Singh et al. [9]. For each outcome variable, several multivariable regression models were run with relevant change categories included in separate models. Not all change categories were expected to have an effect on all four outcomes. For example, the PNC changes would not be expected to have an effect on skilled delivery, and thus regressions with these change categories are not presented for skilled delivery. A separate set of regressions to study the effect of program intensity, defined as the monthly number of change categories tested, was also run. These models also controlled for the independent variables presented earlier.

Due to the amount of missing data and the presence of both serial autocorrelation and clustering, we used generalized estimating equations (GEE) to run the regression analyses. GEE uses all data that is available and assumes data is missing completely at random, which is a plausible assumption for these monthly facility-level data. Autocorrelation and clustering violate the ordinary least squares assumption of uncorrelated error terms, biasing the standard errors when using standard linear regression. GEE is an extension of the quasi-likelihood approach used in generalized linear models and is often applied to modelling longitudinal data [17–19].

In addition, a sensitivity analysis was conducted as a check against our results from the main GEE analysis. In these analyses, single imputation was used to either (1) impute all missing values or (2) impute missing values only for facilities with less than 25% of their observations missing. The imputation was conducted by taking the average value of the nearest non-missing preceding and succeeding values. Because the results of our sensitivity analyses generally corroborated results from the main model, only results for the main model are presented.

The number of observations for each regression model varies slightly by facility type due to the varying amounts of outcome data available. In addition, not all independent variables were available for each facility. Comparisons of mean outcomes for facilities that have all control variables and those that do not were made and found not to be significantly different.

## Results

### Descriptive presentation of the independent variables

All independent variables are presented in Tables 2 and 3. The majority of facilities were health centres (45.5%) and health posts (50.4%), while only 4% were hospitals. Ninety-two percent of the facilities were government affiliated and 8% were faith based. A total of seven project officers were part of the Wave 2 program team, and the average percent of insured patients at a facility was 78%. The PNC change intervention activities were the most common changes implemented in health centres and health posts, while triage was the most common change category in hospitals. Eighty-seven percent of facilities were part of Wave 2a, and 13% were part of Wave 2b.

**Table 2:** Descriptive statistics for control variables and independent variable phase

	n	% or mean
Control variables		
Type of facility		
Hospital	30	4.1
Health centre	334	45.5
Health post	370	50.4
Affiliation of facility		
Government	676	92.1
Faith-based	58	7.9
Number of facilities for each Project Officer		
Project Officer 1	69	10.6
Project Officer 2	110	16.9
Project Officer 3	80	12.3
Project Officer 4	78	12.0
Project Officer 5	63	9.7
Project Officer 6	125	19.2
Project Officer 7	125	19.2
Profession of QI Team Leader		
Midwife	267	51.3
Nurse	119	22.9
Other	134	25.8
Mean number of QI Team Members	NA	9.9
Outpatients with health insurance	NA	78.1
Independent variable		
Phase		
Wave 2a	638	87.1
Wave 2b	96	12.9

*NA* not applicable, *QI* quality improvement

**Table 3:** Descriptive statistics on change category variables, by facility type, n (%)

Change category	Hospitals	Health centres	Health posts
Health education	16 (53.3)		
Targeting primary providers	2 (6.7)		
Triage	25 (83.3)		
Training	7 (23.3)		
Task shifting/Nurse empowerment	12 (40.0)		
Early pregnancy identification		186 (55.7)	160 (43.2)
Four ANC visits		153 (45.8)	154 (41.6)
Skilled delivery/immediate PNC		211 (63.2)	210 (56.8)
PNC day 1 or 2		238 (71.3)	224 (60.5)
PNC day 6 or 7		235 (70.4)	223 (60.3)
Total	30 (100)	335 (100)	370 (100)

*ANC* antenatal care, *PNC* postnatal care

### Descriptive analysis

The comparison of means for the pre-intervention, transition and post-intervention phases is presented in Table 4. Overall, there are improvements in the maternal health outcomes over time. At the aggregate level, there is an increase in early ANC from 37% to 42% to 48% from the pre-intervention phase to the transition phase to the post-intervention phase, respectively. Overall, skilled delivery is at 42% in the pre-intervention phase, increases to 47% in the transition phase and then further increases to 51% in the post-intervention phase. In terms of the child health outcomes, there was an overall increase in the percent of underweight infants attending CWCs, from 2% in the pre-intervention phase to 8% in the post-intervention phase. Under-five mortality decreases from 36/1000 in the pre-intervention phase to 29/1000 in the post-intervention phase.

**Table 4:** Means of the outcome variables by implementation period and facility type

	Pre-intervention			Transition phase			Post-intervention			Overall		
Outcomes	Facility-Months	Mean	Range	Facility-Months	Mean	Range	Facility-Months	Mean	Range	Facility-Months	Mean	Range
Early antenatal care (%)	8832	37	0–100	2077	42	0–100	11,671	48	0–100	22,580	43	0–100
Hospitals	580	40	2–100	117	40	3–94	496	44	3–100	1193	41	2–100
Health centres	5227	36	0–100	1150	41	0–100	6080	45	0–100	12,457	41	0–100
Health posts	3025	38	0–100	810	43	0–100	5095	51	0–100	8930	46	1–100
Skilled delivery (%)	8156	42	0–100	1923	47	0–100	9573	51	0–100	19,652	47	0–100
Hospitals	562	86	0–100	119	89	0–100	416	90	0–100	1097	88	0–100
Health centres	4930	49	0–100	1109	55	0–100	5296	65	0–100	11,335	57	0–100
Health posts	2664	19	0–100	695	27	0–100	3861	28	0–100	7220	25	0–100
Underweight in infants (%)	7482	2	0–100	1942	2	0–67	7685	8	0–100	17,109	5	0–100
Hospitals	213	2	0–27	50	2	0–18	182	6	0–51	445	4	0–51
Health centres	4368	2	0–100	1030	2	0–52	3968	7	0–70	9366	4	0–100
Health posts	2901	2	0–63	862	2	0–67	3535	9	0–100	7298	5	0–100
Under-five mortality rate (Hospitals)	269	36 per 1000	0–100 per 1000	49	25 per 1000	0–23 per 1000	253	29 per 1000	0–39 per 1000	571	32 per 1000	0–100 per 1000

### Time series analysis

#### Health posts

Three of the categories of change interventions – early ANC (β = 0.3540, *P* < 0.01), four or more ANC visits (β = 0.2882, *P* < 0.05) and skilled delivery/immediate PNC (β = 0.2822, *P* < 0.01) – were significantly and positively associated with the skilled delivery outcome (Table 5). Facilities that tested these changes saw 28–35% higher rates of skilled delivery than facilities that did not test such changes. The corresponding trend variables had small positive associations with the skilled delivery outcome, suggesting that the initial positive effect continued over time, but this trend effect was significant only for the early ANC and skilled delivery/immediate PNC changes.

**Table 5:** Results of generalized estimating equation regressions of health outcomes on change categories at health posts

	Early ANC		Skilled delivery			Underweight infants	
	*β* (95% CI)		*β* (95% CI)			*β* (95% CI)	
Wave 2b vs. 2a	−0.0035 (−0.0580 to 0.0510)	0.0080 (−0.0443 to 0.0602)	−0.0163 (−0.1294 to 0.0968)	−0.0281 (−0.1381 to 0.0819)	−0.0268 (−0.1359 to 0.0823)	−0.0049 (−0.0271 to 0.0173)	−0.0044 (−0.0267 to 0.0179)
Early ANC change	0.1316 (−0.0320 to 0.2951)		0.3540 (0.1097 to 0.5983)**				
Early ANC trend	0.0001 (−0.0000 to 0.0003)		0.0003 (0.0000 to 0.0006)*				
Four or more ANC visits change		0.0836 (−0.0769 to 0.2441)		0.2882 (0.0470 to 0.5293)*			
Four or more ANC visits trend		0.0002 (−0.0000 to 0.0003)		0.0002 (−0.0000 to 0.0005)			
Skilled delivery change					0.2822 (0.0671 to 0.4973)*		
Skilled delivery trend					0.0003 (0.0000 to 0.0005)*		
PNC on day 1 or 2 change						0.0667 (0.0209 to 0.1124)**	
PNC on day 1 or 2 trend						0.0000 (−0.0000 to 0.0001)	
PNC on day 6 or 7 change							0.0578 (0.0141 to 0.1015)**
PNC on day 6 or 7 trend							0.0000 (−0.0000 to 0.0001)
Constant	0.2305 (0.1765 to 0.2845)***	0.2233 (0.1693 to 0.2774)***	0.1465 (0.0517 to 0.2413)**	0.1458 (0.0513 to 0.2403)**	0.1348 (0.0394 to 0.2301)**	0.0400 (0.0203 to 0.0597)***	0.0387 (0.0188 to 0.0585)***
n	4902	4902	3923	3923	3923	4154	4154

All models control for project officer, government vs. catholic facility, insurance status, profession of QI team leader, and number of QI team members

*ANC* antenatal care, *PNC* postnatal care

* *P* < 0.05; ** *P* < 0.01; *** *P* < 0.001

In terms of findings for child health, both PNC change categories were significantly associated with a greater percentage of underweight infants among CWC attendees. The β_2_ coefficient was 0.0667 at *P* < 0.01 for the PNC day 1 or 2 change category, and the β_2_ coefficient was 0.0578 at *P* < 0.01 for the PNC day 6 or 7 change category. The corresponding trend variables were not significant.

There was one significant finding for the measure of intensity in the health post analysis, namely the monthly number of change categories tested (Table 6). Intensity was significantly associated with a lower percentage of underweight infants among all CWC attendees (β = –0.0092; *P* < 0.001).

**Table 6:** Results of generalized estimating equation regressions of health outcomes on intensity for health posts and health centres

	Early ANC	Skilled delivery	Underweight infants	Under-five mortality
	*β* (95% CI)	*β* (95% CI)	*β* (95% CI)	*β* (95% CI)
Health posts				
Wave 2b vs. 2a	0.0038 (−0.0486 to 0.0563)	−0.0150 (−0.1248 to 0.0948)	−0.0185 (−0.0443 to 0.0074)	NA
Intensity	−0.0048 (−0.0106 to 0.0010)	0.0066 (−0.0035 to 0.0167)	−0.0092*** (−0.0115 to −0.0068)	NA
Constant	0.2356*** (0.1892 to 0.2821)	0.1942*** (0.1103 to 0.2780)	−0.0320** (−0.0515 to −0.0124)	NA
N	4902	3923	4154	NA
Health centres				
Wave 2b vs. 2a	−0.0047 (−0.0522 to 0.0428)	−0.0047 (−0.1196 to 0.1102)	−0.0244* (−0.0435 to −0.0053)	
Intensity	0.0017 (−0.0026 to 0.0061)	0.0089* (0.0008 to 0.0169)	−0.0088*** (−0.0105 to −0.0071)	
Constant	0.2671*** (0.2317 to 0.3025)	0.3055*** (0.2298 to 0.3812)	–0.0032 (–0.0175 to 0.0110)	
N	6888	6259	5272	

All models control for project officer, government vs. catholic facility, insurance status, profession of QI team leader, and number of QI team members

*ANC* antenatal care

* *P* < 0.05; ** *P* < 0.01; *** *P* < 0.001

### Health centres

None of the specific change categories were associated with the early ANC and skilled delivery outcomes (Table 7). Both PNC change categories, however, were associated with an increased percent of underweight infants among attendees at CWCs (β = 0.0701, *P* < 0.001 and β = 0.0452, *P* < 0.001, respectively). The trend variables were significant and positive, indicating that the initial increased effect was maintained. Wave 2b was significantly and negatively associated with attendance of underweight infants at CWCs in the models with the PNC change categories.

**Table 7:** Results of generalized estimating equation regressions of health outcomes on change categories at health centres

	Early ANC		Skilled delivery			Underweight Infants	
	*β* (95% CI)		*β* (95% CI)			*β* (95% CI)	
Wave 2b vs. 2a	0.0016 (–0.0466 to 0.0497)	–0.0068 (–0.0542 to 0.0407)	0.0079 (–0.1083 to 0.1241)	–0.0180 (–0.1334 to 0.0975)	–0.0112 (–0.1255 to 0.1032)	–0.0195 (–0.0364 to –0.0026)*	0.0196 (–0.0366 to –0.0026)*
Early ANC change	–0.0519 (–0.1456 to 0.0418)		0.0045 (–0.1593 to 0.1683)				
Early ANC trend	–0.0001 (–0.0002 to 0.0000)		0.0000 (–0.0002 to 0.0002)				
Four or more ANC visits change		–0.0676 (–0.1468 to 0.0116)		0.0136 (–0.1318 to 0.1590)			
Four or more ANC visits trend		–0.0001 (–0.0002 to 0.0000)		0.0000 (–0.0002 to 0.0002)			
Skilled delivery change					–0.0317 (–0.1685 to 0.1052)		
Skilled delivery trend					0.0000 (–0.0002 to 0.0002)		
PNC on day 1 or 2 change						0.0701 (0.0436 to 0.0967)***	
PNC on day 1 or 2 trend						0.0001 (0.0000 to 0.0001)***	
PNC on day 6 or 7 change							0.0452 (0.0216 to 0.0688)***
PNC on day 6 or 7 trend							0.0000 (0.0000 to 0.0001)**
Constant	0.2526 (0.2133 to 0.2919)***	0.2538 (0.2142 to 0.2935)***	0.2753 (0.1928 to 0.3579)***	0.2688 (0.1834 to 0.3501)***	0.2724 (0.1885 to 0.3563)***	0.0425 (0.0281 to 0.0568)***	0.0425 (0.0278 to 0.0571)***
N	6888	6888	6259	6259	6259	5272	5272

All models control for project officer, government vs. catholic facility, insurance status, profession of QI team leader, and number of QI team members

*ANC* antenatal care, *PNC* postnatal care

* *P* < 0.05; ** *P* < 0.01; *** *P* < 0.001

There were two significant associations between the measure of intensity and the outcome variables (Table 6). A greater number of change categories tested was significantly associated with increased skilled delivery (β = 0.0089, *P* < 0.05) and a smaller percentage of underweight infants among child wellness attendees (β = –0.0088, *P* < 0.001). There was a significant association in the intensity models between Wave 2b and decreased underweight infants at CWCs.

### Hospitals

There were several significant associations between the change categories in the regressions for hospitals (Tables 8 and 9). Facilities that tested a health education change had 58% higher rates of skilled delivery compared to facilities not testing this change category (β = 0.5753, *P* < 0.05). The trend variable was slightly positive and significant, indicating that the increase in skilled delivery was continued although at a lower level than the initial increase. The triage change category was associated with a 42% increase in early ANC (β = 0.4236, *P* < 0.05) and a 50% increase in skilled delivery (β = 0.4989, *P* < 0.05), and the trend variable for the latter indicated a slight but significant increase over time (β = 0.0004, *P* < 0.05). Across all outcomes, there were no significant associations with the implementation phase variable in the hospital settings.

**Table 8:** Results of generalized estimating equation regressions of maternal health outcomes on change categories at hospitals

	Early ANC					Skilled delivery				
	β (95% CI)					β (95% CI)				
Wave 2b vs. 2a	0.0000 (0.0000 to 0.0000)	0.0000 (0.0000 to 0.0000)	0.0000 (0.0000 to 0.0000)	0.0000 (0.0000 to 0.0000)	0.0000 (0.0000 to 0.0000)	0.0000 (0.0000 to 0.0000)	0.0000 (0.0000 to 0.0000)	0.0000 (0.0000 to 0.0000)	0.0000 (0.0000 to 0.0000)	0.0000 (0.0000 to 0.0000)
Health education change	0.3535 (–0.0397 to 0.7468)					0.5753 (0.0743 to 1.0763)*				
Health education trend	0.0002 (–0.0001 to 0.0005)					0.0005 (0.0001 to 0.0008)*				
Targeting primary providers change		0.0000 (0.0000 to 0.0000)					–3.3742 (–26.4808 to 19.7323)			
Targeting primary providers trend		0.0000 (0.0000 to 0.0000)					–0.0032 (–0.0225 to 0.0161)			
Triage change			0.4236 (0.0440 to 0.8031)*					0.4989 (0.0159 to 0.9819)*		
Triage trend			0.0002 (–0.0001 to 0.0005)					0.0004 (0.0001 to 0.0008)*		
Training change				0.5092 (–0.0439 to 1.0624)					–0.0142 (–0.6329 to 0.6045)	
Training trend				0.0004 (–0.0001 to 0.0008)					0.0000 (–0.0004 to 0.0005)	
Task shifting/nurse empowerment change					0.2949 (–0.1446 to 0.7345)					0.2800 (–0.2507 to 0.8107)
Task shifting/nurse empowerment trend					0.0001 (–0.0003 to 0.0004)					0.0003 (–0.0001 to 0.0007)
Constant	0.1668 (0.0480 to 0.2857)**	0.1649 (0.0467 to 0.2832)**	0.1650 (0.0477 to 0.2822)**	0.1717 (0.0538 to 0.2896)**	0.1667 (0.0509 to 0.2824)**	1.0583 (0.9345 to 1.1821)***	1.0816 (0.9583 to 1.2048)***	1.0669 (0.9441 to 1.1897)***	1.0744 (0.9514 to 1.1975)***	1.1076 (0.9837 to 1.2316)***
n	570	570	570	570	570	574	574	574	574	574

All models control for project officer, government vs. catholic facility, insurance status, profession of QI team leader, and number of QI team members

*ANC* antenatal care

* *P* < 0.05; ** *P* < 0.01; *** *P* < 0.001

**Table 9:** Results of generalized estimating equation regressions of child health outcomes on change categories at hospitals

	Underweight infants					Under-five mortality				
	β (95% CI)					β (95% CI)				
Wave 2b vs. 2a	0.0000 (0.0000 to 0.0000)	0.0000 (0.0000 to 0.0000)	0.0000 (0.0000 to 0.0000)	0.0000 (0.0000 to 0.0000)	0.0000 (0.0000 to 0.0000)	0.0000 (0.0000 to 0.0000)	0.0000 (0.0000 to 0.0000)	0.0000 (0.0000 to 0.0000)	0.0000 (0.0000 to 0.0000)	0.0000 (0.0000 to 0.0000)
Health education change	0.0241 (–0.2843 to 0.3324)					0.0528 (–0.1509 to 0.2565)				
Health education trend	0.0001 (–0.0002 to 0.0003)					0.0001 (–0.0001 to 0.0002)				
Targeting primary providers change		0.0000 (0.0000 to 0.0000)					0.8415 (–0.3567 to 2.0396)			
Targeting primary providers trend		0.0000 (0.0000 to 0.0000)					0.0006 (–0.0002 to 0.0013)			
Triage change			0.0241 (–0.2843 to 0.3324)					0.0798 (–0.1135 to 0.2731)		
Triage trend			0.0001 (–0.0002 to 0.0003)					0.0000 (–0.0001 to 0.0002)		
Training change				0.2223 (–0.1437 to 0.5883)					0.1525 (–0.3240 to 0.6289)	
Training trend				0.0002 (–0.0001 to 0.0005)					0.0001 (–0.0002 to 0.0005)	
Task shifting/nurse empowerment change					0.2558 (–0.1129 to 0.6246)					–0.0539 (–0.2073 to 0.3151)
Task shifting/nurse empowerment trend					0.0002 (–0.0001 to 0.0005)					0.0000 (–0.0002 to 0.0002)
Constant	0.0412 (–0.1164 to 0.1987)**	0.0406 (–0.1177 to 0.1989)	0.0412 (–0.1164 to 0.1987)	0.0099 (–0.1492 to 0.1691)	0.0220 (–0.1367 to 0.1808)	0.0687 (0.0232 to 0.1142)**	0.0687 (0.0296 to 0.1079)***	0.0542 (0.0092 to 0.0093)*	0.0761 (0.0308 to 0.1215)***	0.0678 (0.0197 to 0.1159)**
n	226	226	226	226	226	284	284	284	284	284

All models control for project officer, government vs. catholic facility, insurance status, profession of QI team leader, and number of QI team members

* *P* < 0.05; ** *P* < 0.01; *** *P* < 0.001

Greater intensity was significantly associated with two of the outcomes in hospitals (Table 10). Intensity was associated with a 0.9% decrease in underweight infants attending CWCs (β = –0.0093, *P* < 0.01) and a 0.4% decrease in under five-mortality (β = –0.0038, *P* < 0.05). Once again there were no significant associations for the implementation phase variable.

**Table 10:** Results of generalized estimating equation regression of health outcomes on intensity for hospitals

	Early ANC	Skilled delivery	Underweight infants	Under-five mortality
	β (95% CI)	β (95% CI)	β (95% CI)	β (95% CI)
Wave2b vs. 2a	0.0000 (0.0000 to 0.0000)	0.0000 (0.0000 to 0.0000)	0.0000 (0.0000 to 0.0000)	0.0000 (0.0000 to 0.0000)
Intensity	0.0073 (–0.0044 to 0.0189)	–0.0005 (–0.0137 to 0.0127)	–0.0093** (–0.0201 to 0.0014)	–0.0038* (–0.0090 to 0.0013)
Constant	0.1773** (0.0601 to 0.2945)	1.0985*** (0.9772 to 1.2197)	–0.0815 (–0.2571 to 0.0942)	0.0527* (0.0052 to 0.1002)
N	570	574	226	284

*ANC* antenatal care

## Discussion

As more low- and middle-income countries implement QI projects to improve health outcomes, there is a need to evaluate the approaches both during pilot and scale-up phases. Evaluations of pilot phases can help demonstrate the evidence needed to justify scale-up and/or can provide valuable information to inform implementation modifications for the scale-up phase [20]. Due to the magnitude of scale-up phases and the difficulty of finding control or comparison groups, innovative evaluation approaches are needed that can take advantage of existing monitoring data [21,22]. In this paper, we present an innovative evaluation of the scale-up phase of ‘Project Fives Alive!’ using data from Ghana’s routine health information system supplemented by facility characteristics and program records.

Findings from the evaluation indicated a positive effect of ‘Project Fives Alive!’ on key maternal and child health outcomes. There was evidence of some sustained program effect on underweight infants attending CWCs and skilled delivery as was seen in Wave 1 [9]. All the maternal health-focused change categories were associated with an increase in skilled delivery for health posts, and the health education and triage change categories were associated with the early ANC and skilled delivery outcomes for hospitals. Greater intensity was associated with increased skilled delivery for health centres.

There were positive effects of the PNC change categories in getting more underweight infants into care in health posts and health centres; however, greater intensity was also negatively associated with the percentage of underweight infants at CWCs across the facility types. These differing findings need a nuanced explanation. It could be that the PNC change categories initially increased care-seeking of caregivers of underweight infants. Over time, as these facilities implemented more changes and more fully incorporated the QI approach into their daily work, there could have been overall improvements in the health and nutrition of children in the catchment area, leading to a lower percentage of children in facilities who were underweight. These findings on underweight children are important given that under-nutrition is estimated to be an underlying factor in 45% of under-five deaths [23].

In Wave 1, there were no significant associations between the change categories or intensity with mortality. Perhaps due to the longer time period of Wave 2 compared to Wave 1 (51 months versus 21 months) we see evidence of impact on mortality for Wave 2. In hospitals, greater intensity was associated with slightly decreased under-five mortality. As health providers engaged more fully in the QI approach over time, they may have been able to improve the quality of services provided and/or increase early care-seeking such that mortality declined.

There were few significant differences by phase of implementation, indicating that all facilities benefited from the intervention. In ‘Project Fives Alive!’ the first set of Wave 2a reached full saturation in March 2010 compared to March 2011 for the first set of Wave 2b facilities. This is an important finding given that scale-up strategies of many projects need to have a phased approach to attain broad reach.

There are several limitations to this analysis, including our inability to study population-level mortality. We only had data on facility deaths and not deaths that occurred in communities. In Ghana, as in many low- and middle-income countries, many under-five deaths occur at home or in non-facility environments. An additional data challenge is that we could only study skilled delivery until December 2011 for a large number of facilities because of the change in reporting. Finally, it is difficult to find comparison groups for the evaluation of a scale-up phase of a project, and our analysis lacked such groups. We were able to use each facility as its own control in an interrupted time series analysis with additional control for potential confounding factors, including program and facility characteristics. We also controlled for National Health Insurance Scheme registration, which also has a strong focus on maternal and child health. The use of repeated monthly outcome data from each facility, both pre- and post-intervention, offers a strong evaluation design [16]. We cannot, however, completely rule out the possibility that other ongoing maternal and child health initiatives could have also influenced the results.

## Conclusion

Findings from the scale-up phase of ‘Project Fives Alive!’ indicate program effects on the key maternal and child health outcomes studied, including reduced under-five mortality. The QI approach of identifying barriers to care and care-seeking with local, simple and inexpensive solutions has demonstrated impact at scale and should be considered a feasible approach for improving maternal and child health outcomes in other low- and middle-income settings. We also demonstrate the feasibility of using existing outcome data in a multivariable time series analysis to evaluate the scale-up phase of an intervention.

## Abbreviations

ANC, Antenatal care; DHIMS, District Health Information Management System; GEE, Generalized estimating equations; MDG, Millennium Development Goal; PNC, Postnatal care; QI, Quality improvement
